# Supplementary material for: Characterization of a new Leishmania major strain for use in a controlled human infection model
Source: Nat Commun. 2021 Jan 11;12:215. doi: 10.1038/s41467-020-20569-3 (PMC7801518; doi:10.1038/s41467-020-20569-3)
Supplement: Supplementary file 1 — Supplementary Information File [file 41467_2020_20569_MOESM1_ESM.pdf]

Characterization of a new *Leishmania major* strain for use in a controlled human infection model. Ashwin et.al

Supplementary Information

a

Chromosome	Location	Reference nucleotide	Alternative Nucleotide	Freq	Gene	Notes
**Post-Infection – Polymorphism Gained**						
LmjF.29	1072606	A	AG	29.41% (Het)	Intergenic region	Homopolymer stretch
LmjF.36	2505068	C	CT	35.37% (Het)	Intergenic region	Homopolymer stretch
LmjF.30	114857	A	ATG	35.93% (Het)	Intergenic region	Homopolymer stretch

b

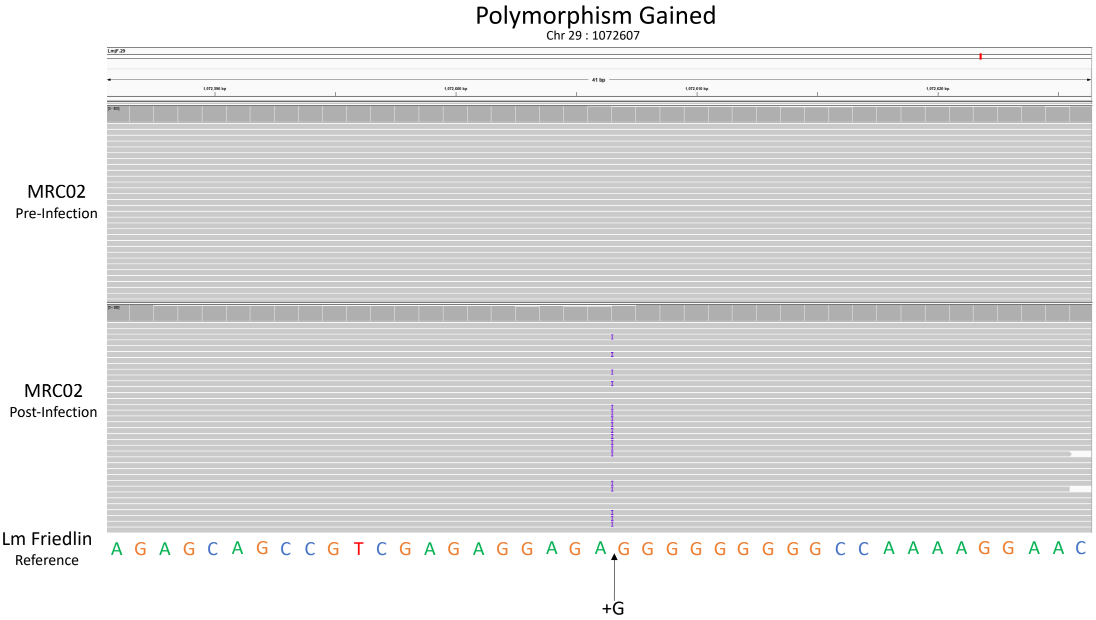

Supplementary Figure 1 Polymorphisms between *L. major* MRC-02 before and after mouse passage

a Specific location and details of each polymorphism. Note that all the changes were in homopolymer stretches in non-coding regions and were in less that 50% of the reads.
b Example of polymorphism gained on the Chromosome 29, location 1072607 where a G nucleotide was inserted in the post-infection genome in 29% of the reads as indicated.

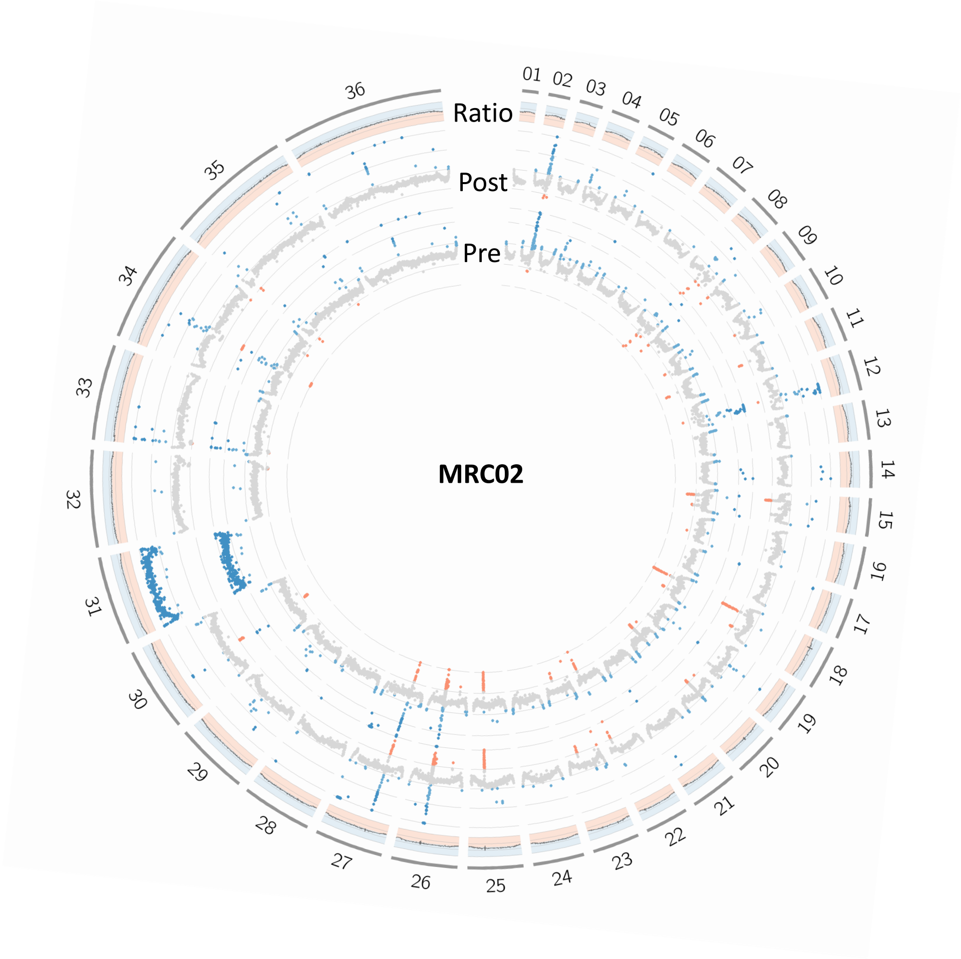

Supplementary Figure 2. Gene copy number variations over chromosomes 1 to 36.

Raw sequencing depth per gene across all chromosomes plotted on a scale of 0-1500 and color-coded according to lower (red), median (grey), or higher (blue) read depth (Pre and Post rings). Note that chromosome 31 is tetraploid (Blue) in *L. major* while all other chromosomes are normally diploid. Coverage change ratio between the BALB/c passage (Post) and cultured (Pre) *L. major* MRC-02 parasites shown in the outer ring on a scale of 0 to 2.

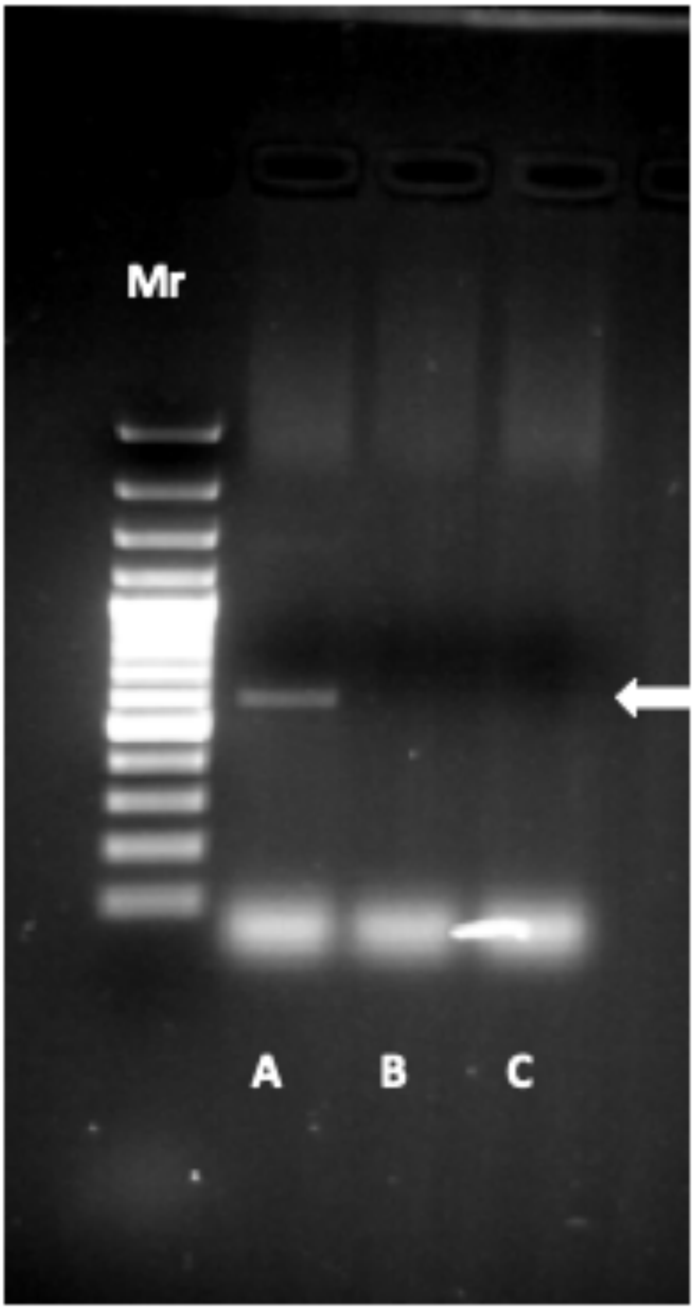

Supplementary Figure 3. Analysis of LRV2 presence in *Leishmania* strains by RT-PCR.

RNA was purified from each isolate and reverse transcribed into cDNA. Specific PCR targeting the RNA - dependent RNA polymerase (*RdRp*) gene of LRV2 was carried out. Arrow indicates 526 bp product. Mr - 100 bp molecular weight marker. Lane A - *L. aethiopica* LRC-L494 positive control; Lane B - *L. major*_MRC01; *L. major*_MRC02. Data are derived from a single experiment.

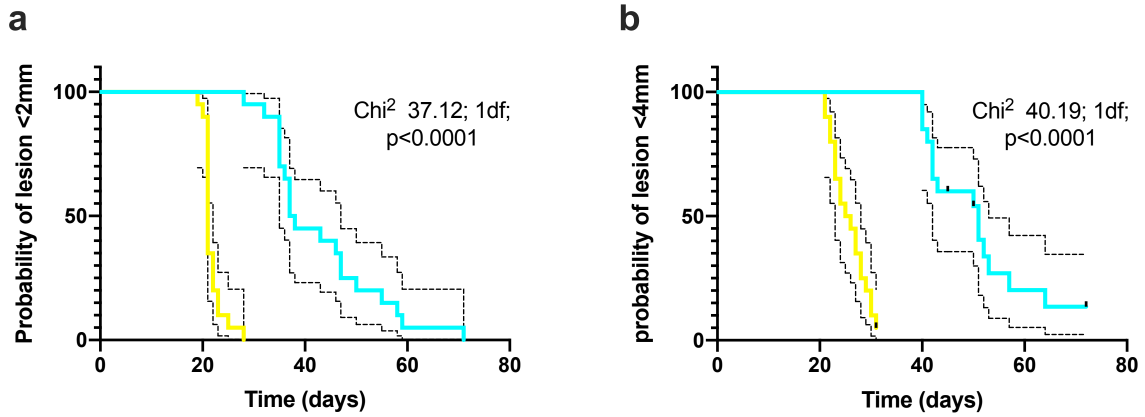

Supplementary Figure 4. Rate of lesion development for *L. major* MRC-01 and *L. major* MRC-02 in BALB/c mice after needle challenge

Time to event (lesion size) is shown as Kaplan Meier plots for mice infected with *L. major* MRC-01 (n=20, blue) and *L. major* MRC-02 (n=20, yellow). **a** lesion of >2mm. **b** lesion of >4mm. Data were analysed using the Log-rank (Mantel-Cox) test and a two tail p value calculated. Dotted lines represent 95% CI. Median times to lesion > 2mm were 37.5 and 21.0 days, for *L. major* MRC-01 and *L. major* MRC-02 respectively (ratio 1.786, 95% CI of ratio 0.96 to 3.32; p<0.0001). Median times to lesion >4mm were 51.0 vs 25.5 for *L. major* MRC-01 and *L. major* MRC-02 respectively (ratio 2.0, 95% CI of ratio 1.02 to 3.94; p<0.0001).

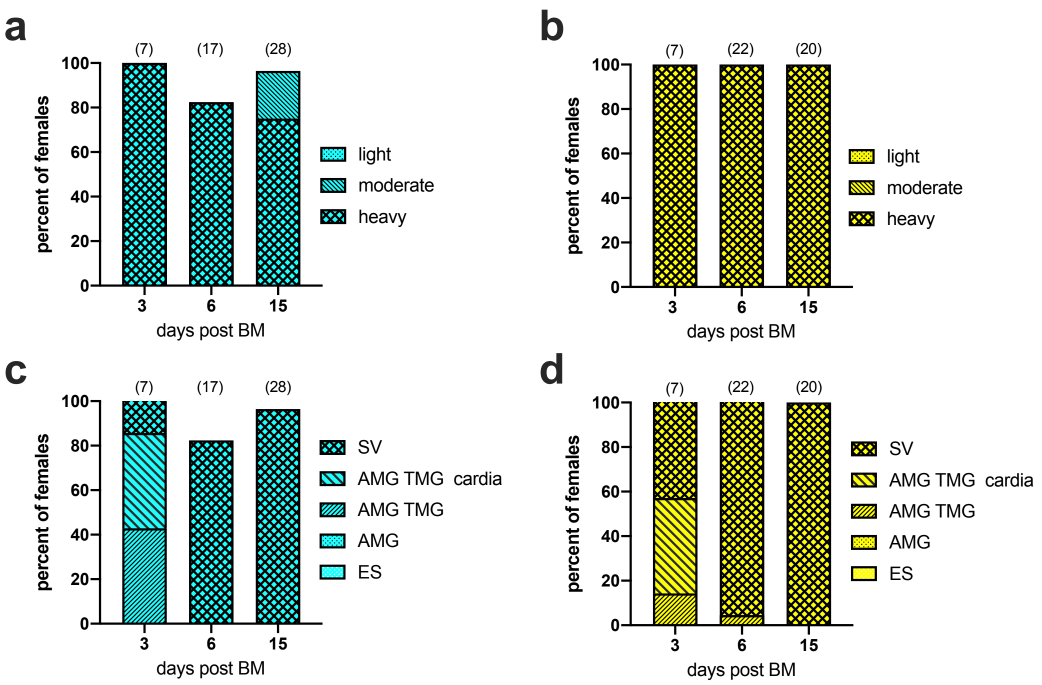

Supplementary Figure 5. Sand fly development of parasites recovered from Research Banks

Infection rates and intensity of infections in *P. duboscqi* females infected with *L. major* MRC-01 (**a**) and *L. major* MRC-02 (**b**). Localisation of infections in *P. duboscqi* females infected with *L. major* MRC-01 (**c**) and *L. major* MRC-02 (**d**). Number of sand flies dissected is shown above each bar.

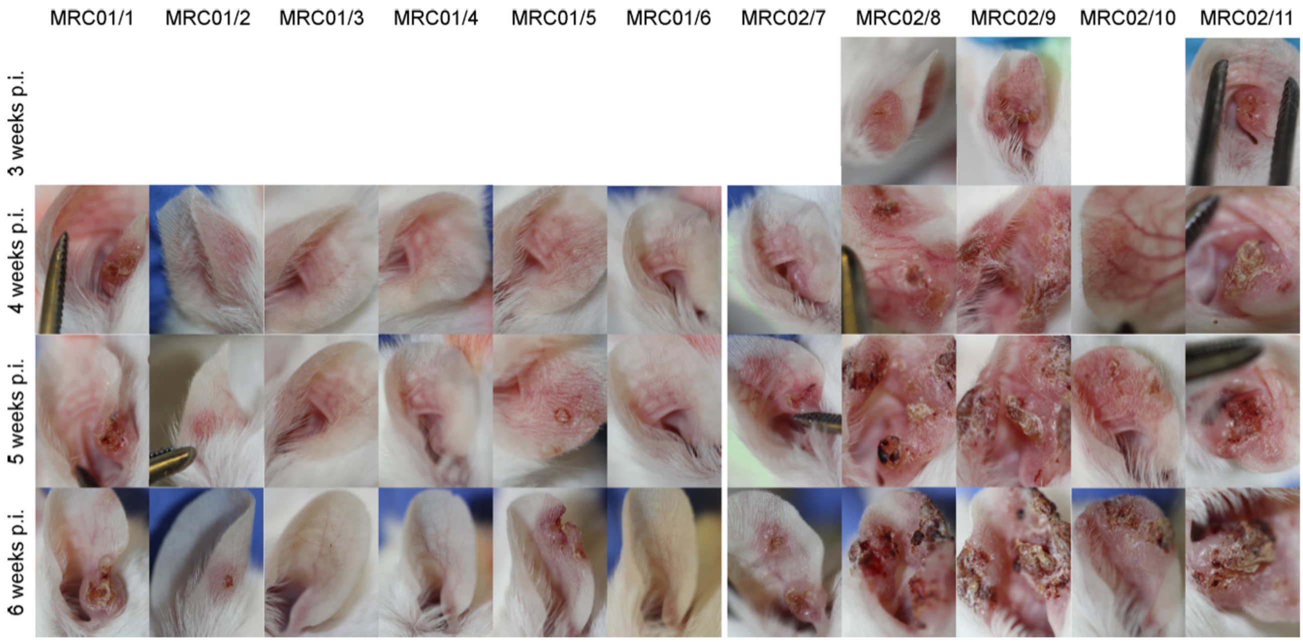

Supplementary Figure 6. Development of lesions in BALB/c mice after exposure to infected sand fly bites.

Images show ears photographed at weeks 3-6 post exposure to 10 *P. duboscqi* infected with *L. major* MRC-01 (n=6) and *L. major* MRC-02 (n=5).

Isolate	Sample	No sand flies	feeding rate*	No. parasite posiitve females**	No. parasites per ear	Mean no. parasites per engorged sand fly	Mean No. parasites / all infected sand flies
MRC-01	1	10	70%	4+1	0	0	0
	2	10	60%	2+3	143	72	29
	3	10	0	0+4	70	0	18
	4	10	40%	3+2	7240	2413	1448
	5	10	50%	2+4	506	253	84
	6	10	20%	2+6	708	354	89
	**Total**	**60**	**40%**	**13+20**	**8667**	**667**	**270**
MRC-02	1	10	50%	4+2	3060	765	510
	2	10	40%	1+3	576	576	144
	3	10	90%	8+1	5670	709	630
	4	10	50%	2+1	333	167	111
	5	10	70%	1+2	92	92	31
	6	10	80%	2+1	106	53	35
	**Total**	**60**	**63%**	**18+10**	**9837**	**546**	**351**

* Feeding rate = No. of engorged females / total No.

L. major isolate	Mouse no.	No. of used sandf fly females	Feeding rate (No. of engorged females/total	No. of Leishmania positive females (fed+unfed)	First swelling appearance (weeks p.i.)	First lesion appearance (weeks p.i.)
MRC-01	1	10	60%	4 + 2	3	4
	2	10	40%	2 + 5	4	5
	3	10	70%	4 + 1	-	-
	4	10	80%	4 +0	-	-
	5	10	100%	5 + 0	3	4
	6	10	50%	2 + 2	-	-
MRC-02	7	10	70%	3 + 1	3	4
	8	10	50%	5 + 5	2	3
	9	10	70%	6 + 1	2	3
	10	10	70%	5 + 2	3	3
	11	10	70%	2 + 1	-	4

Supplementary Table 1. Research Bank infections post bite in BALB/c mice

Table shows parasite load per ear determined by qPCR post bite with 10 infected sand flies (top; sheet 1) and fly feeding rates and rate of lesion development (bottom; sheet 2).
