# Supplementary material for: Characterization of a new Leishmania major strain for use in a controlled human infection model
Source: Nat Commun. 2021 Jan 11;12:215. doi: 10.1038/s41467-020-20569-3 (PMC7801518; doi:10.1038/s41467-020-20569-3)
Supplement: Supplementary file 2 — Description of Additional Supplementary Files [file 41467_2020_20569_MOESM2_ESM.pdf]

Description of Additional Supplementary Files

File Name: Supplementary Data 1
Description: Source data for and additional data supporting Figure 6.
Each tab indicates numbers of sand flies, species of infecting parasite and stages of parasite development for all time points analysed. Dub, *P. duboscqi*; pap, *P. papatasi*; BM, blood meal.
