# Supplementary material for: Characterization of a new Leishmania major strain for use in a controlled human infection model
Source: Nat Commun. 2021 Jan 11;12:215. doi: 10.1038/s41467-020-20569-3 (PMC7801518; doi:10.1038/s41467-020-20569-3)
Supplement: Supplementary file 4 — Reporting Summary [file 41467_2020_20569_MOESM4_ESM.pdf]

nature research

Corresponding author(s): Paul Kaye

Last updated by author(s): Nov 30, 2020

Reporting Summary

Nature Research wishes to improve the reproducibility of the work that we publish. This form provides structure for consistency and transparency in reporting. For further information on Nature Research policies, see our Editorial Policies and the Editorial Policy Checklist.

Statistics

For all statistical analyses, confirm that the following items are present in the figure legend, table legend, main text, or Methods section.

n/a	Confirmed	
☐	☒	The exact sample size (*n*) for each experimental group/condition, given as a discrete number and unit of measurement
☐	☒	A statement on whether measurements were taken from distinct samples or whether the same sample was measured repeatedly
☐	☒	The statistical test(s) used AND whether they are one- or two-sided<br>*Only common tests should be described solely by name; describe more complex techniques in the Methods section.*
☒	☐	A description of all covariates tested
☐	☒	A description of any assumptions or corrections, such as tests of normality and adjustment for multiple comparisons
☐	☒	A full description of the statistical parameters including central tendency (e.g. means) or other basic estimates (e.g. regression coefficient) AND variation (e.g. standard deviation) or associated estimates of uncertainty (e.g. confidence intervals)
☐	☒	For null hypothesis testing, the test statistic (e.g. *F*, *t*, *r*) with confidence intervals, effect sizes, degrees of freedom and *P* value noted<br>*Give P values as exact values whenever suitable.*
☒	☐	For Bayesian analysis, information on the choice of priors and Markov chain Monte Carlo settings
☒	☐	For hierarchical and complex designs, identification of the appropriate level for tests and full reporting of outcomes
☐	☒	Estimates of effect sizes (e.g. Cohen's *d*, Pearson's *r*), indicating how they were calculated

Our web collection on statistics for biologists contains articles on many of the points above.

Software and code

Policy information about availability of computer code

Data collection	No software was used for data collection
Data analysis	The Burrows Wheeler Aligner 70 (version 0.7.17), VarScan2 71 (version 2.4.3) , samtools (version 1.10), circos (veersion 0.69-6) and Integrative Genomics Viewer (version 2.7.1) were used for analysis of parasite genome data. These software packages are referenced. Statistical software packages SPSS version 23 and GraphPad Prism 8 for macOS (v8.4.2) were used.

For manuscripts utilizing custom algorithms or software that are central to the research but not yet described in published literature, software must be made available to editors and reviewers. We strongly encourage code deposition in a community repository (e.g. GitHub). See the Nature Research guidelines for submitting code & software for further information.

Data

Policy information about availability of data

All manuscripts must include a data availability statement. This statement should provide the following information, where applicable:

- Accession codes, unique identifiers, or web links for publicly available datasets
- A list of figures that have associated raw data
- A description of any restrictions on data availability

Data availability statement: publicly available data
Parasite genomics data used in compiling Figure 2 are available from TriTrypDB (https://tritrypdb.org/tritrypdb/app).

Data availability statement: raw data
Sequence data for L. major MRC-01 and L. major MRC-02 are available from GenBank (BioProject PRJNA633113, https://www.ncbi.nlm.nih.gov/bioproject/PRJNA633113: accession numbers SAMN14933143, https://www.ncbi.nlm.nih.gov/sra/SRX8423856[accn]; SAMN14933144, https://www.ncbi.nlm.nih.gov/sra/

SRX8423855[accn] and SAMN14933145, https://www.ncbi.nlm.nih.gov/sra/SRX8423854[accn]). Source data and additional data supporting Figure 6 are provided in Supplementary Data 1. Other source data are provided as a Source Data file.

Combined data availability statement (manuscript)

Sequence data for L. major MRC-01 and L. major MRC-02 are available from GenBank (BioProject PRJNA633113, https://www.ncbi.nlm.nih.gov/bioproject/PRJNA633113: accession numbers SAMN14933143, https://www.ncbi.nlm.nih.gov/sra/SRX8423856[accn]; SAMN14933144, https://www.ncbi.nlm.nih.gov/sra/SRX8423855[accn] and SAMN14933145, https://www.ncbi.nlm.nih.gov/sra/SRX8423854[accn]). Parasite genomics data used in compiling Figure 2 are available from TriTrypDB (https://tritrypdb.org/tritrypdb/app). Parasites produced under GMP will be available for clinical assessment of candidate Leishmania vaccines under an appropriate MTA. Source data and additional data supporting Figure 6 are provided in Supplementary Data 1. Other source data are provided as a Source Data file.

Field-specific reporting

Please select the one below that is the best fit for your research. If you are not sure, read the appropriate sections before making your selection.

- [x] Life sciences
- [ ] Behavioural & social sciences
- [ ] Ecological, evolutionary & environmental sciences

For a reference copy of the document with all sections, see nature.com/documents/nr-reporting-summary-flat.pdf

Life sciences study design

All studies must disclose on these points even when the disclosure is negative.

Sample size	No formal sample size calculations were performed as this was a comparative observational study with no specific hypothesis under test. Sample sizes for transmission and drug treatment studies were comparable to or exceed previously published studies (e.g. Rogers et al. Transmission of cutaneous leishmaniasis by sand flies is enhanced by regurgitation of fPPG, Nature, 2004. 430: 463- 46; Wijnant, G. J., et al Efficacy of Paromomycin-Chloroquine Combination Therapy in Experimental Cutaneous Leishmaniasis. Antimicrobial agents and chemotherapy, 2017. 61(8), e00358-17).
Data exclusions	One mouse died from unknown causes (unrelated to infection) and was excluded from analysis.
Replication	All mouse experiments were performed at least twice and data are shown pooled for both experiments. Analysis of data from individual expts showed similar trends.
Randomization	For drug treatments, mice reaching a pre-determined cut-off of 4mm were randomized (n=9-10 per group) to receive either saline or paromomycin (50mg/kg, i.p daily for 10 days). Sand fly transmission studies in mice were not randomised.
Blinding	Mice were evaluated for lesion progression and parasite growth blinded to treatment group and parasite strain. Sand fly parasite development studies were blinded to parasite strain.

Reporting for specific materials, systems and methods

We require information from authors about some types of materials, experimental systems and methods used in many studies. Here, indicate whether each material, system or method listed is relevant to your study. If you are not sure if a list item applies to your research, read the appropriate section before selecting a response.

Materials & experimental systems

n/a	Involved in the study	
x		Antibodies
x		Eukaryotic cell lines
x		Palaeontology and archaeology
	x	Animals and other organisms
	x	Human research participants
	x	Clinical data
x		Dual use research of concern

Methods

n/a	Involved in the study	
x		ChIP-seq
x		Flow cytometry
x		MRI-based neuroimaging

Animals and other organisms

Policy information about studies involving animals; ARRIVE guidelines recommended for reporting animal research

Laboratory animals	Adult female specific pathogen free BALB/c mice between 8-12 weeks old were used in all experiments reported here and were obtained from either AnLab s.r.o (Prague) or Charles River UK (York). Mice were maintained in individually ventilated cages with food and water ad libitum and a 12 h light/12 h dark photoperiod in rooms maintained at 56% humidity and 20-21oC.<br><br>The colonies of P. duboscqi and P. papatasi (originating in Senegal and Turkey, respectively) were maintained in the insectary of the Department of Parasitology, Charles University in Prague, under standard conditions (26°C on 50 % sucrose, humidity in the insectary 60-70% and 14 h light/10 h dark photoperiod) as described previously 75. The sand fly colonies have been screened by RT-PCR and found to be negative for Phleboviruses (including Sandfly Fever Sicilian Virus group, Massilia virus and Toscana Virus) and Flaviviruses (targeting a conserved region of the NS5 gene). Only female sand flies are used in experiments and were fed blood meals at 5-9 days of age..
Wild animals	No wild animals were used in this study
Field-collected samples	No field collected animals were used in this study.
Ethics oversight	Animals were maintained and handled at Charles University and the University of York in accordance with institutional guidelines and national legislation (Czech republic: Act No. 246/1992 and 359/2012 coll. on Protection of Animals against Cruelty in present statutes at large; UK: Animals (Scientific Procedures) Act 1986). All the experiments were approved by: i) the Committee on the Ethics of Laboratory Experiments of the Charles University in Prague and were performed under permit from the Ministry of Education, Youth and Sports of the Czech Republic (MSMT-28321/2018-6), and ii) The University of York Animal Welfare and Ethics Review Board and performed under Home Office license (PPL P49487014).

Note that full information on the approval of the study protocol must also be provided in the manuscript.

Human research participants

Policy information about studies involving human research participants

Population characteristics	Two individuals (female, 41 years of age; male, 22 years of age) from non-endemic areas of central Israel that had self-referred to Sheba Hospital in early 2019 after developing lesions subsequent to visiting the endemic region of Negev served as parasite donors.
Recruitment	The two parasite donors self referred for treatment and were diagnosed with CL by PCR. They consented to for samples to be taken for the purposes of this study and parasite cultures grew aseptically. There was no bias in patient selection for the purposes of the current study which does not relate to clinical parameters.
Ethics oversight	All human studies were conducted in accord with the Declaration of Helsinki. Ethical approval was obtained from the Helsinki Committees of Hebrew University (0400-18-SOR) and The Chaim Sheba Medical Centre (5658-18-SMC) and the University of York Dept. of Biology Ethics Committee. Informed consent was obtained from patients with PCR confirmed leishmaniasis for parasite isolation and subsequent use of these parasites in the development of a human challenge model.

Note that full information on the approval of the study protocol must also be provided in the manuscript.

Clinical data

Policy information about clinical studies

All manuscripts should comply with the ICMJEguidelines for publication of clinical research and a completedCONSORT checklist must be included with all submissions.

Clinical trial registration	This study does not involve a clinical trial. A related clinical trial to assess feasibility of sand fly biting protocols in humans using uninfected sand flies is found at www.clinicaltrials.gov; NCT03999970;
Study protocol	To be appended as supplementary
Data collection	The associated clinical trial NCT03999970 is a healthy-volunteer clinical study to develop a sand fly biting protocol. 12 participants were recruited for the study. It was an open-label randomized clinical study with 2 arms (for 2 different species of sand fly: Phlebotomus papatasi and Phlebotomus duboscqi). Participants were randomized to each arm with 6 participants in each arm. This study took place at the Translational Research Facility (Q Block), Hull York Medical School & Department of Biology, University of York, York between Oct 2019 and Jan 2020.<br>The participants will be followed up to 21 days post-sand fly bite. There will then be a focus group once all participants have completed their follow-up.
Outcomes	For the associated clinical trial NCT03999970<br><br>Primary Outcome Measures :<br>Percentage of participants who successfully undergo sand fly bite during a 30 minute exposure determined by visual dermatoscopy

[Time Frame: 21 days]
Development of a sand fly biting protocol using pathogen-free sand flies which is effective and safe for volunteers:
Volunteers aged between 18-65 years will receive a bite or bites by sand flies using a watch-like biting chamber placed on the arm. The investigators will initially evaluate the use of biting chambers containing up to 5 sand flies maintained on the arm for 30 minutes, and evaluate the two sand fly species Phlebotomus papatasi and Phlebotomus duboscqi fed on blood twice in the laboratory prior to human exposure.

Secondary Outcome Measures :
Percentage of participants with visual changes following sand fly bite using photography. [Time Frame: 21 days]
The investigators will use photography to identify and record skin changes following sand fly bite by use of photography.

Percentage of participants with visual changes following sand fly bite using dermatoscopy. [Time Frame: 21 days]
The investigators will use dermatoscopy to identify and record skin changes following sand fly bite.

Percentage of participants with demonstrable serological evidence of sand fly bite. To determine human immunological response to sand fly bite using serology and cellular response measurement. [Time Frame: 21 days]
The investigators will measure serology including Leishmania and sand fly salivary gland antibodies following sand fly bite.

Percentage of participants with demonstrable cellular response evidence of sand fly bite. [Time Frame: 21 days]
The investigators will measure cellular response following sand fly bite

Percentage of participants with demonstrable evidence of change in IgE (immunoglobulin E) following sand fly bite. [Time Frame: 21 days]
The investigators will measure IgE at baseline and following sand fly bite

Percentage of participants with significant change in C-reactive protein following sand fly bite. [Time Frame: 21 days]
C-Reactive protein will be measured at baseline and following sand fly bite/

Determine size of lesion following sand fly bite over time [Time Frame: 21 days]
Rulers will be used to determine the changing size of any lesion
